# Male and Female Differences in Homicide Mortality: Results of an Italian Longitudinal Study, 2012–2018

**DOI:** 10.3389/fpubh.2022.919335

**Published:** 2022-07-13

**Authors:** Martina Ventura, Anteo Di Napoli, Alessio Petrelli, Marilena Pappagallo, Concetta Mirisola, Luisa Frova

**Affiliations:** ^1^Department of Epidemiology, National Institute for Health, Migration and Poverty (INMP), Rome, Italy; ^2^Directorate for Social Statistics and Welfare, Italian National Institute of Statistics (Istat), Rome, Italy

**Keywords:** homicide, mortality, sex, health outcomes, cohort study

## Abstract

**Introduction:**

Italy has one of the lowest homicide rates in Europe. However, while it is decreasing overall, the proportion of murdered women is increasing. This study aimed to analyze the demographic and socioeconomic characteristics associated with homicide mortality in Italy, focusing specifically on male and female differences.

**Methods:**

Using a longitudinal design, the Italian 2011 General Census population was followed up to 2018. Deaths from homicide were retrieved by a record linkage with the Causes of Death Register. Age-standardized mortality rates, stratified by sex, citizenship, education, and geographic area of residence were calculated. The association between sociodemographic characteristics and homicide mortality was evaluated using quasi-Poisson regression models.

**Results:**

Between 2012 and 2018, 1,940 homicides were recorded in Italy: 53% were females over age 55, 10% were immigrant females, 34% were males aged 40–54 years, 76% had a medium-low education level, and 57% lived in the South and Islands. Foreign citizenship increased a female's risk of dying from homicide (adjusted rate ratio (RRadj): 1.85; 95% CI: 1.54–2.23), while no differences between Italian and immigrant males were found. An inverse association between education and mortality was observed for both sexes, stronger for males (RRadj: 3.68; 95% CI: 3.10–4.36, low vs. high) than for females (RRadj: 1.38; 95%CI: 1.17–1.62, low vs. high). Moreover, a male residing in the South or the Islands had almost 2.5 times the risk of dying from homicide than a resident in the North-West. Finally, old age (over 75) increased a female's risk of being murdered, whereas the highest risk for males was observed for those aged 25–54 years.

**Conclusions:**

Male and female differences in homicide mortality profiles by age were expected, but the results by residence, citizenship, and education highlight that living in disadvantaged socioeconomic contexts increases the risk of dying from homicide, suggesting the need to implement specific prevention and intervention strategies.

## Introduction

Homicide is an extreme expression of violence and a premature cause of death. In 2017, about 464,000 people worldwide were victims of homicide (global rate: 6.1 per 100,000 inhabitants) (1, 2).

Rates in high-income countries/areas are generally lower than rates in low-income and middle-income countries or areas. About 80% of homicides occur in males, with the highest rates in males aged 15–29 years (3, 4).

In Italy, the homicide rate is lower than in other European countries, with an incidence of 0.53 per 100,000 inhabitants in 2019 (1). The European average incidence was 0.93 per 100,000; the phenomenon has been decreasing overall in recent years, although the reduction mainly concerns males, who started at higher rates, than it does females (5).

Various circumstances, motivations, and relationships can act as driving forces of homicide, and they are often overlapping and multifaceted. The Global Study on Homicide of the United Nations Office on Drugs and Crime classifies homicide into three main typologies: homicide related to interpersonal conflict, homicide related to criminal activities, and homicide related to sociopolitical agendas (2).

The violence of all types is strongly associated with social determinants; the combination of unemployment, low income, gender inequalities, and limited educational opportunities can negatively affect the social climate, generating violence (3).

In Europe, differences in homicide rates across countries can be explained mostly in terms of their respective level of socioeconomic development. Policies aimed at achieving improvements in wealth, education, and other crucial development areas would be expected to push homicide rates down. Moreover, factors such as alcohol abuse or progress in gender equality can also play an important role in increasing or decreasing rates, respectively (2).

It has been frequently highlighted that male gender, young-adult age, low socioeconomic status, and being involved in organized crime are the main risk factors for being victims of homicide (6).

However, the characterization of the risk of homicide should take into account the different contexts in which murders of males and females take place. In fact, there are two structurally different phenomena: females are mainly killed at home by partners or family members, while males are killed in public spaces by criminals (1, 2).

Although women and girls account for a far smaller overall share of victims of homicide than do men, they bear by far the greatest burden of intimate partner/family-related homicide (2). A specific definition was conceived for the term *femicide*: “the killing of a woman by an intimate partner and the death of a woman as a result of a practice that is harmful to women.” The term is based on gender inequalities and a gender-based reason for murder (7). In other words, the concept of femicide centers on the characteristics of both the victim and the perpetrator and is, according to the Istanbul Convention, the murder of a woman (by a man) because she is a woman (8).

In Italy, due to the absence of specific legislation, it is not yet easy to statistically identify the variables that help to detect femicides based on these definitions. However, in an attempt to identify them, it was observed in 2019 that around 84% of female murders in our country could be classified as femicides (1).

A systematic review concerning the period 1990–2011 found that globally, an estimated 13.5% of homicides were committed by an intimate partner, a proportion was six times higher for female homicides than for male homicides, with the highest percentages in high-income countries (9). In Italy, in 2019, 61.3% of female homicides were committed by a partner or ex-partner, 6% more than in 2018 (1, 5).

Exploring differences in homicide characteristics of males and females could provide evidence-based support for prevention policies of public safety authorities, to define targeted prevention interventions, in particular, to reduce domestic violence against females.

In recent years, the Italian National Institute of Statistics (Istat) has developed a national follow-up system based on the cohort generated by the record linkage between the Causes of Death and the Population and Housing Census archives. The record linkage makes it possible to explore demographic and socioeconomic inequalities in general and cause-specific mortality (10–12).

This study aimed to longitudinally analyze demographic and socioeconomic characteristics associated with homicides in the Italian population recorded in the 2011 General Census and followed up until 2018. The analysis focused on male and female differences.

## Methods

### Data Sources, Study Population, and Design

The study was conducted on the population cohort conceived within the project “Socioeconomic differences in mortality” (IF IST 2646), part of the National Statistical Program (PSN) approved by the Italian Data Protection Authority (13). Using a retrospective longitudinal design, the individuals recorded in the 2011 General Census and residents in Italy, considered the initial cohort, were followed up from 2012 to 2018, until death, emigration, or end of the follow-up, whichever came first, yielding a maximum of 7 years of follow-up. Information on mortality was retrieved from the Istat National Causes of Death Register, which annually collects all deaths occurring in Italy. The Resident Population Register, which records the cancellations of people due to death, emigration to another municipality or abroad, and unavailability from the census or ascertained, was used to identify exit from the cohort for emigration. These 3 registers were connected through a record linkage using the fiscal code (a unique personal identification number issued to all residents in Italy at birth or upon immigration) as the linkage key. The reliability of the fiscal code was very high in all the registers, making it possible to link 97.1% of all deaths among the census population occurring in Italy in the period 2012–2014 (11). Since there is no reason to believe that the reliability of the fiscal code reported in the 3 registers decreased over subsequent years, the performance of the record linkage is expected to be equally high.

Emigration flows are used to define the person-years in the cohort follow-up. As the emigration abroad is at first autonomously communicated at the local Resident Population Register, there might be an underestimate of outflows, differently between Italian and foreign citizens. However, municipalities currently carry out activities to update their local Resident Population Register, and Istat statistics already contain these updates.

All the individuals at least 15 years of age on 1 January 2012 were included in the study population. The choice of the lower age limit was applied as we assumed that homicides happening in childhood have different dynamics and specificities that deserve a separate discussion (14).

### Outcome and Determinants

The present study analyses homicide mortality between 2012 and 2018. Among all deaths, homicides were selected according to the rules and provisions included in the International Classification of Diseases, 10th Revision (ICD-10: X85-Y09, Y87.1).

Sex, age, citizenship, education level, and residence area variables were used to characterize the cohort and as potential demographic and socioeconomic factors affecting homicide mortality. Citizenship was used to define the status of immigrants, dichotomized in Italian and foreign (or stateless). Italy is a country with a recent migratory tradition, where migratory flows have become significant over the last 25 years. To obtain Italian citizenship is linked to very restrictive constraints, based on the ius sanguinis: all immigrants remain with their native citizenship, except for those who marry an Italian or ask for citizenship after a period ranging between 3 and 10 consecutive years of legal residence. Moreover, children born in Italy to foreign parents can obtain citizenship after their 18th birthday. Therefore, all non-Italian citizens have had a migration path to Italy and therefore can be considered immigrants.

Age groups were defined to have balanced population groups, and to highlight any patterns in the youngest (under 25) and the elderly (over 75), thus maintaining a sufficient number of subjects (at least 10% of the population) in these classes.

Education was categorized into three levels: high (secondary school or university degree), medium (middle school), and low (elementary school or none). The 20 Italian regions were aggregated in five geographic macro areas: North-West (Piedmont, Valle d'Aosta, Lombardy, Liguria), North-East (Trentino Alto Adige, Veneto, Friuli Venetia Giulia, Emilia-Romagna), Center (Tuscany, Umbria, Marche, and Latium), South (Abruzzo, Molise, Campania, Apulia, Basilicata, and Calabria), and Islands (Sicily and Sardinia).

### Statistical Analysis

To assess male and female differences in homicide mortality, all the statistical analyses were performed separately for males and females.

Baseline sociodemographic characteristics of the cohort and the deaths occurring during follow-up are described; crude mortality rates per 100,000 person-years were calculated. Age-standardized mortality rates per 100,000 person-years were calculated, by citizenship, education level, and geographic macro area; applied weights were the proportion of individuals in each age group in the 2013 European Standard Population (15).

To quantify the differences in mortality in relative terms, the ratios between age-standardized mortality rates were computed separately (mortality rate ratios, MRRs) by citizenship, education level, and area, considering “Italians”, “high education,” and “North-West,” respectively, as reference categories (16). The associated 95% CIs of rates and rate ratios were calculated.

The effect of age, citizenship, education level, and residence area on homicide mortality was evaluated using a multivariate quasi-Poisson regression model for overdispersed count data with log link function, stratified by sex (17). Adjusted rate ratios with 95% CIs were estimated.

All analyses were performed using SAS^®^ System version 9.3.

## Results

At the beginning of the follow-up (1 January 2012), the study population included 51,034,816 subjects aged 15 years or older (Supplementary Appendix). The sociodemographic characteristics of the cohort at the beginning of the follow-up and the deaths occurring during the study period are shown in Table 1. In Italy between 2012 and 2018, 1,940 homicides were recorded with an average follow-up of 3.18 and 3.36 years for males and females, respectively. In total, 35% of the victims were female and 8% had foreign citizenship and almost half lived in the South or the Islands. When comparing deaths of males and females by age, we observed a higher proportion of female homicides in older ages (52.7% over age 55, of whom 27.4% over age 75 years), while 34.2% of males aged 40–54 were murdered. The proportion of immigrants who died from homicide was higher among females than males (10.5% and 6.5%, respectively). As for education, the highest share of homicides among males was observed for those with medium education level, and among females with low or high education level. Moreover, 28.8% of the women killed were residents in the North-West of Italy, while 57.4% of the men killed resided in the South or the Islands.

**Table 1:** Characteristics of the cohort at the beginning of follow-up and of the deaths, by sex, 2012–2018.

**Homicides**	**Females**				**Males**				*p*-value*
	**Cohort (*N*)**	**%**	**Deaths**	**%**	**Cohort (*N*)**	**%**	**Deaths**	**%**	
**Total**	26,608,287	*52.1*	678	34.9	24,426,529	47.9	1,262	65.1	
**Characteristics**
**Age**
15–24	2,875,993	*10.8*	38	*5.6*	3,028,074	*12.4*	111	*8.8*	<0.0001
25–39	5,776,149	*21.7*	114	*16.8*	5,749,010	*23.5*	303	*24.0*	
40–54	6,962,082	*26.2*	169	*24.9*	6,769,070	*27.7*	431	*34.2*	
55–74	7,177,892	*27.0*	171	*25.2*	6,526,562	*26.7*	312	*24.7*	
75+	3,816,171	*14.3*	186	*27.4*	2,353,813	*9.6*	105	*8.3*	
**Citizenship**
Italian	24,860,678	*93.4*	607	*89.5*	22,971,160	*94.0*	1,180	*93.5*	<0.05
Foreign/stateless	1,747,609	*6.6*	71	*10.5*	1,455,369	*6.0*	82	*6.5*	
**Education level**
High	11,920,076	*44.8*	239	*35.3*	11,243,128	*46.0*	300	*23.8*	<0.0001
Medium	7,469,193	*28.1*	195	*28.8*	8,640,236	*35.4*	628	*49.8*	
Low	7,219,018	*27.1*	244	*36.0*	4,543,165	*18.6*	334	*26.5*	
**Residence area**
North-West	7,090,801	*26.6*	195	*28.8*	6,506,757	*26.6*	215	*17.0*	<0.0001
North-East	5,116,364	*19.2*	137	*20.2*	4,726,758	*19.4*	127	*10.1*	
Center	5,269,078	*19.8*	142	*20.9*	4,759,658	*19.5*	196	*15.5*	
South	6,172,656	*23.2*	132	*19.5*	5,703,799	*23.4*	482	*38.2*	
Islands	2,959,388	*11.1*	72	*10.6*	2,729,557	*11.2*	242	*19.2*	

^*^P values of the comparison of deaths by sex. For the row “Total” in italics are reported the row percentages. For the other rowsare reported the column percenages.

Overall, a crude homicide rate of 0.55 per 100,000 person-years was observed, 0.74 for males and 0.37 for females. Crude and age-standardized rates were systematically higher in males than in females, for all the characteristics of interest (Table 2). An increased risk of dying from homicide was observed for immigrant females compared to Italian females (MRR: 1.82; 95% CI: 1.22–2.73), while no differences by citizenship were found for men. A lower education level was associated with a higher risk of homicide mortality in both sexes, but especially for males (MRR: 5.64; 95% CI: 4.44–7.15, low vs. high education). Moreover, while the macro area of residence was not associated with homicide mortality in females, a male residing in the South or the Islands had almost 2.5 times the risk of dying from homicide than a resident in the North-West.

**Table 2:** Homicide mortality: crude and age-standardized rates *100,000 and mortality rate ratios (MRR) with 95% CI, by citizenship, education level, and residence area.

**Homicides**	**Females**					**Males**				
	Crude rate*100,000	Age Std Rate*100,000	**Age Std Rate 95%CI**	**MRR**	**MRR 95%CI**	Crude rate*100,000	Age Std Rate *100,000	**Age Std Rate 95%CI**	**MRR**	**MRR 95%CI**
**Total**	0.367	0.290	(0.269–0.314)			0.741	0.618	(0.585–0.653)		
**Characteristics**										
**Citizenship**										
Italian	0.352	0.272	(0.251–0.295)	1	*Ref*	0.738	0.617	(0.583–0.654)	1	*Ref*
Foreign/stateless	0.569	0.496	(0.335–0.741)	1.82	(1.22–2.73)	0.781	0.501	(0.390–0.646)	0.81	(0.63–1.05)
**Education level**										
High	0.291	0.299	(0.235–0.379)	1	*Ref*	0.391	0.325	(0.271–0.389)	1	*Ref*
Medium	0.368	0.342	(0.295–0.396)	1.14	(0.86–1.51)	1.037	0.881	(0.812–0.956)	2.71	(2.23–3.31)
Low	0.491	0.493	(0.359–0.676)	1.65	(1.11–2.45)	1.013	1.830	(1.566–2.138)	5.64	(4.44–7.15)
**Residence area**										
North-West	0.398	0.310	(0.270–0.358)	1	*Ref*	0.476	0.392	(0.342–0.449)	1	*Ref*
North-East	0.387	0.309	(0.259–0.367)	1.00	(0.79–1.25)	0.387	0.321	(0.269–0.382)	0.82	(0.66–1.02)
Center	0.390	0.301	(0.253–0.357)	0.97	(0.78–1.21)	0.593	0.494	(0.429–0.569)	1.26	(1.04–1.53)
South	0.305	0.248	(0.209–0.294)	0.80	(0.64–1.00)	1.202	0.998	(0.912–1.091)	2.55	(2.16–2.99)
Islands	0.349	0.287	(0.227–0.362)	0.92	(0.70–1.22)	1.268	1.048	(0.924–1.189)	2.67	(2.22–3.22)

**Figure 1:**
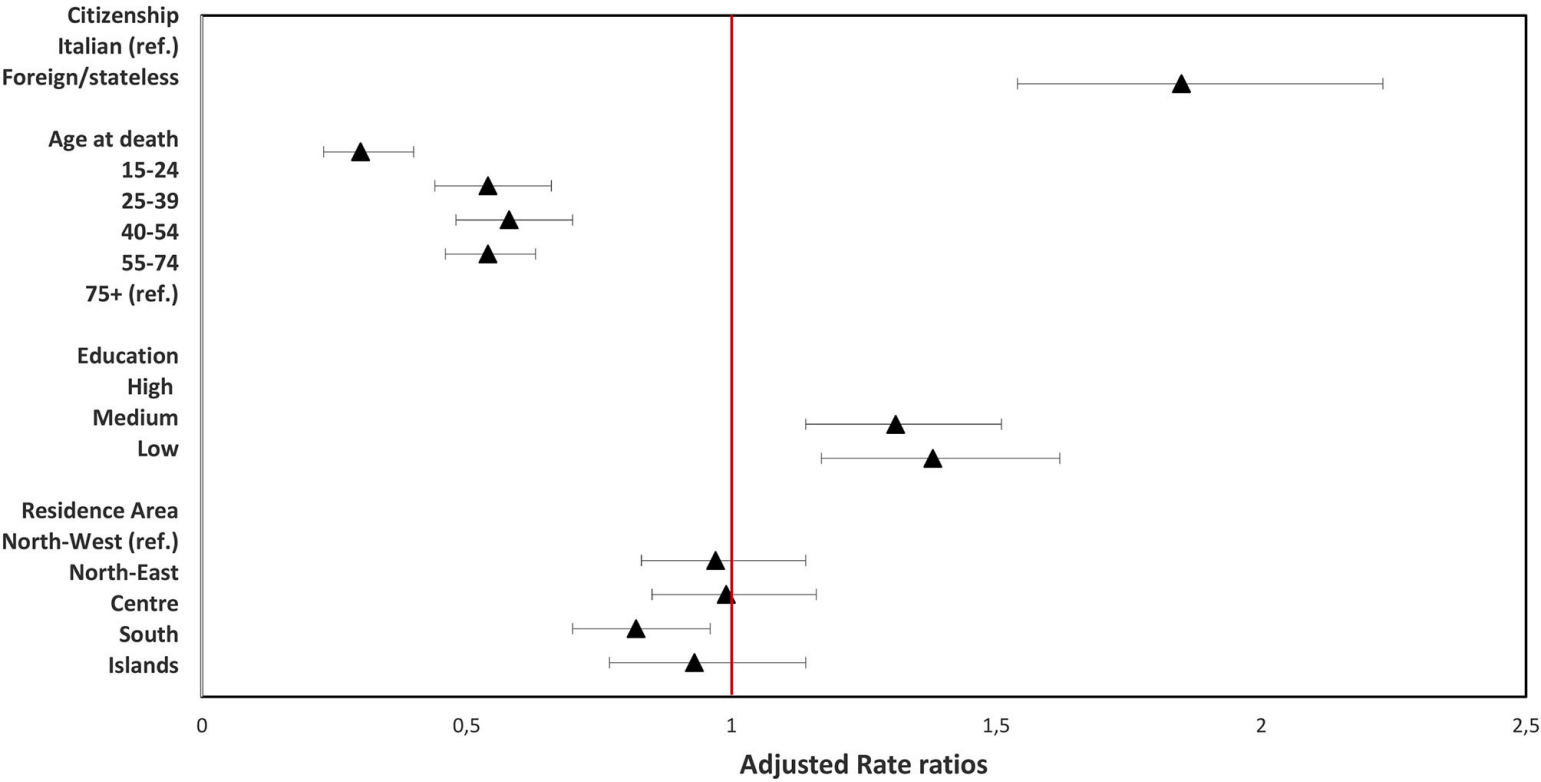
Results of multivariate quasi-Poisson regression model (Females).

**Figure 2:**
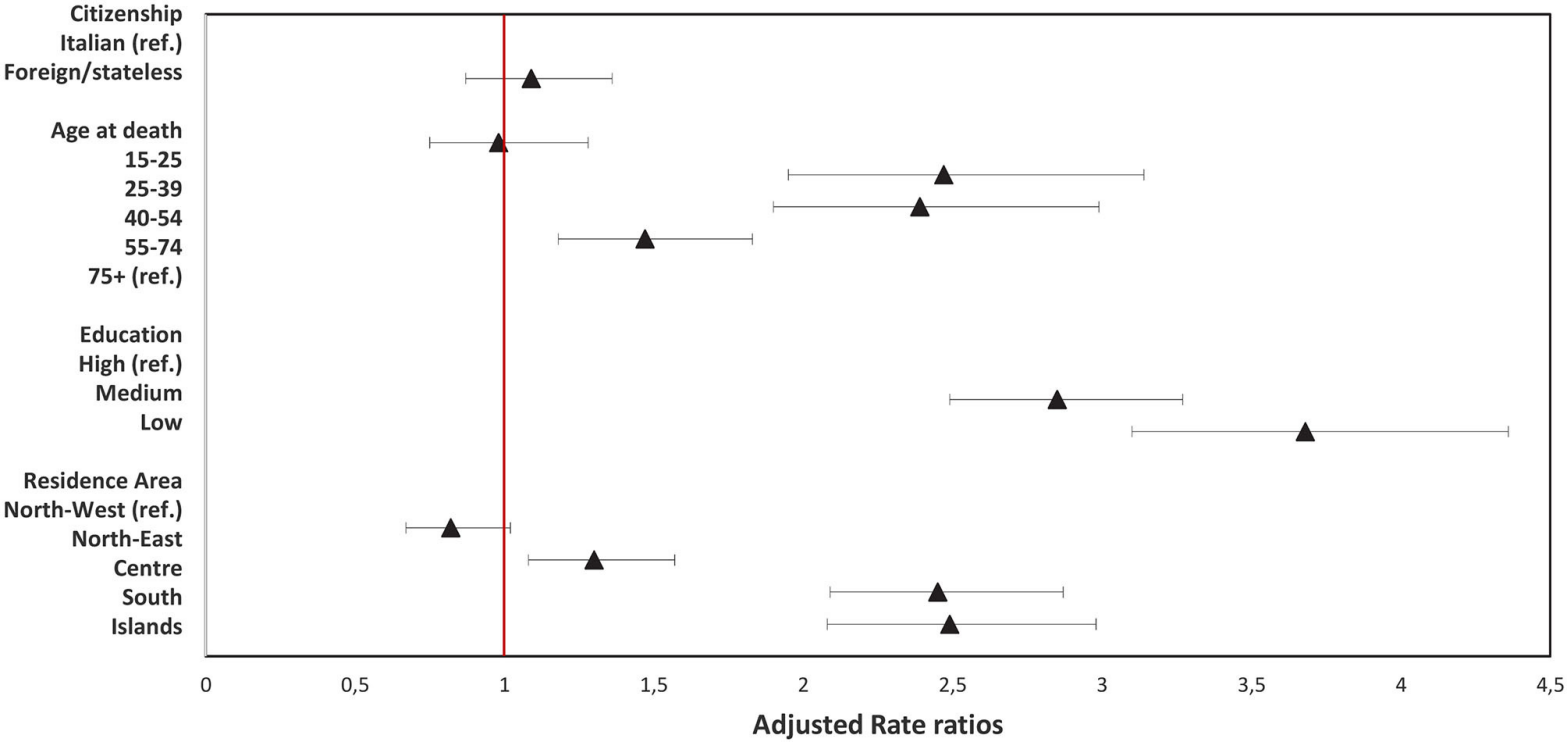
Results of multivariate quasi-Poisson regression model (Males).

Figures 1, 2 show the results of the multivariate regression models for females and males, respectively, in which the effect of age, citizenship, education level, and macro area of residence on homicide mortality was jointly evaluated. When adjusting for all the other conditions, the higher risk of homicide mortality for foreign females compared to Italian females was confirmed (adjusted rate ratio (RRadj): 1.85; 95% CI: 1.54–2.23). The highest risk of dying from homicide was found for females aged over 75 years, and higher mortality risk was also observed in females with a medium or low education level, compared with those with a high level (RRadj: 1.38; 95% CI: 1.17–1.62). A slight protective effect of the macro area of residence was observed for females residing in the South of Italy (RRadj: 0.82; 95% CI: 0.70–0.96, South vs. North-West). The results show a significantly different pattern for males, for whom homicide mortality was higher for those aged 25–54 years, with a risk of homicide more than double that of the ≥75 years age class. Moreover, a strong inverse association was found between education level and mortality, with the highest risk of homicide in males with the lowest education level (RRadj: 3.68; 95% CI: 3.10–4.36, low vs. high). Finally, a geographic gradient was observed in mortality by macro area of residence, with an increased risk of moving from the North to the South and the Islands (RRadj: 2.49; 95% CI: 2.08–2.98, Islands vs. North-West).

## Discussion

This study aimed at evaluating male and female differences in the demographic and socioeconomic characteristics associated with homicide mortality, which could be affected by contextual and cultural factors. To our knowledge, this is the first nationwide longitudinal study investigating male and female differences in socioeconomic and geographic risk factors for homicide mortality in the national cohort of residents in Italy, with a 7-year follow-up.

Our results show that females who had a higher risk of dying from homicide were foreign, older, or had a low or medium education level. For males, the risk of homicide was much higher among those aged 25–54 years, those with a medium or low education level, and those who reside in the South or Islands.

Italy is today one of the safest countries in the world in terms of the risk of being a victim of intentional homicide (18). The crude mortality rate observed for our cohort is in line with that reported by Istat in 2019: that for males is about double that for females (1). In Italy, by comparing current data with those of the 1990s, when there were six times more homicides (1991 was the peak year for homicides), a downward trend in homicides can be seen, describing a phenomenon that has significantly changed over time. Indeed, if in the past almost 40% of murders were attributable to mafia-type organizations, organized crime, although still a phenomenon to be monitored very carefully, is now a less relevant cause for the number of homicides, accounting for less than 10% of these deaths. However, the decrease in organized and unorganized crime-related homicides mainly affected males, in particular those residing in the regions of the South or Islands (1, 19). Conversely, the decrease seen for females was lower and mainly attributable to a reduction in the number of victims killed by unknown perpetrators rather than in intimate partner/family-related homicides (5).

In 2019, 88.3% of female homicides in Italy were committed by a known person. Specifically, more than 60% of the women were killed by their current or previous partner, 22.5% by a family member (including children and parents), and 4.5% by another person the woman knew (friend and colleague). Instead, only 35.7% of male homicides were committed by a known person (including only 5.4% by a partner or ex-partner) (5). In addition, with regard to perpetrators, 13.7% of those accused of voluntary manslaughter in the context of a violent personal relationship are charged with aggravating circumstances “against an ascendant or descendant,” 5.2% “against relatives,” and 1.5% of “sexual assault” (1).

The risk of homicide can be influenced by factors that are more prevalent in immigrant communities such as social isolation, cultural attitudes, gender roles, and fewer employment options (6). In our study, we found a higher risk of homicide among immigrant females, regardless of other characteristics, whereas no differences by citizenship were observed for males. The different patterns by sex among the immigrant population warrants further exploration, however, as, it can be assumed that the intersection between cultural factors and aspects related to the social, economic, and political contexts could explain the higher homicide rates of immigrant females than of Italian females, especially in terms of intimate femicides (20, 21). There may be factors that make female immigrants more vulnerable to violence, including low levels of community support, the absence of appropriate services, cultural views on gender, fear of deportation or of losing custody of children, and legal factors (22, 23). Moreover, female immigrants may also encounter more barriers to accessing support services in case of difficulty, such as living in a violent family context. Research has established that intimate partner violence is a crucial precursor to intimate femicide (24, 25).

Consistently to other studies (25, 26), the older women in our cohort, in particular those over 75 years of age, were more at risk of dying from homicide than younger ones. It has been described that about 50% of murders of women over age 65 are committed by a partner or ex-partner, 30% by a relative, and the remaining 20% by unknown persons (1). Many factors contribute to older women's increased disadvantage and vulnerability to violence (27). Studies on older female victims of domestic or intimate partner homicide have shown that significant fatal risk factors are physical and mental illness, depression, breakdown, and mercy killing, as is being financially dependent on the abusive partner can be a key barrier to seeking help (25, 28–30).

On the other hand, males at the highest risk of dying from homicide were those aged 25–54 years. This is consistent with the greater male involvement in the contexts of unorganized and organized crime. In fact, most male homicides in these age classes are committed by unknown or unidentified persons (1, 30).

In both sexes, education level was negatively associated with homicide mortality. Socioeconomic inequalities in health have been extensively observed in all European countries, frequently using education as an individual proxy of socioeconomic status (31, 32). Concerning mortality from homicide, a recent meta-analysis identified low education level as a risk factor for intimate partner homicide of females and male perpetrators (33). As discussed above for foreign citizenship, low education can lead to a greater vulnerability to violence, lower levels of community support, a lack of appropriate services, and difficulty in accessing support services, resulting in a higher risk of homicide. Indeed, education is positively related to employability, determining a positive impact on the wellbeing of the entire community. In a broader sense, education increases an individual's sense of responsibility, citizenship, and social participation, all factors that can determine a crime-reducing effect (34–38). The fact that murders of males occur more frequently in crime-related contexts can explain a stronger negative effect of education on mortality from this cause; instead, femicides are committed mainly in the domestic context, where education could play a less pronounced role. In fact, our study found that a lower education level led to an ~30% mortality increase in females, whereas less educated males' risk of dying from homicide was more than three times higher.

The macro area of residence also proved to be an important determinant of mortality from homicide for males but not for females. We found that a male living in the South or Islands of Italy had a 2.5 times higher risk than that of a resident in the North, with a North-South gradient of increasing mortality. A previous analysis of the temporal trend up to 2010 described a decrease in homicides for both sexes, more marked in the South and the Islands, with an inversion of the relationship between the geographic areas, with the North-West assuming a greater weight for females than the South and Islands (19). More recently, Istat described that males residing in the North-East of Italy and females living in the South had the lowest rates of homicide, although the geographic differences were very limited (1). The result confirmed what a previous Italian study found (39) and is consistent with the higher concentration of organized crime in the South and Islands of Italy.

Thanks to the powerful information source developed by Istat, this study provides a comprehensive assessment of the inequalities in mortality in Italy across the entire population, with high-quality follow-up data. The use of this data source allowed us to address the issue of diversity, as it was inherent in the objective of the work, by evaluating socioeconomic and geographic inequalities in homicide mortality between individuals with different education levels, citizenship, and area of residence. Moreover, by assessing how demographic and socioeconomic factors affect males' and females' risk of homicide differently, the role of sex in the association under study was also analyzed.

Our study does have some limitations. Although the long follow-up made it possible to identify a sufficiently large number of deaths from homicide, the low incidence of the mortality from this cause in our country results in a low number of deaths, especially when stratifying by multiple variables. This issue mainly concerns females (for example, younger females or those living in the Islands) and the analysis of citizenship for both sexes. Nevertheless, the excess risk observed for immigrant females is considerable and statistically significant. Another limitation of this study is that, due to the type of data source used to enroll the study cohort, it was not possible to analyze the differences in mortality from homicide also by gender. In fact, the Italian General Census allowed us to study the entire Italian population, with a significant gain in dimension and power of the study; however, the General Census only reports information on sex (male and female). In the current context, if we had been able to analyze the difference in homicide by gender, thereby including the entire LGBTQ2 community, this would have contributed to making the results of the study even more valuable, with a gain in terms of knowledge and inclusiveness. Future studies using current data sources should endeavor to address this limitation.

Lastly, the retrospective design of the cohort does not permit updating baseline information over the follow-up or the analysis of other potential exposures and confounders not collected in the census (11). This could represent a limitation when studying the relationship between education and mortality, especially for the younger age groups. Furthermore, some people could have moved to change their residence during the follow-up time, resulting in misclassification of residence at the time of death. Anyway, in this study we considered the geographic macro areas of residence (groups of regions), thus reducing the possible misclassification. Indeed, in Italy, between 2012 and 2018, about 2–2.5% of the population annually changed their residence moving within Italy. However, 70–90% of these transfers take place within the same “macro area” of residence (e.g., from a region of North West to another region of North West) (40). Moreover, when comparing the differences between the “macro area” of residence in the 2011 Census and the same information at death, we observed that the 99% of all deaths and the 98% of homicide deaths maintained the same “macro area” of residence.

## Conclusions

Understanding the differences between females and males in terms of homicide mortality represents a way to explore one dimension of gender-based health inequalities, contributing to the discussion and knowledge of diversity in terms of social inequalities in health. Our study pointed out how the characteristics of the murders differ considerably by sex. Some of these differences in mortality profiles were expected, but the disparities observed in residence, citizenship, and education suggest the presence of subgroups of females and males who are more exposed to the risk of being killed in disadvantaged socioeconomic contexts. Public safety authorities generally focus their homicide prevention strategies on reducing organized and unorganized crime, which is reflected in a reduction in the number of murdered males (41). Conversely, less attention is given to interventions aiming at preventing domestic violence, in particular against females, one of the main risk factors of femicide (24), or at reducing direct access to firearms (33). Our study highlights the need for more targeted prevention interventions. The excess mortality of immigrant females or those with a low education level suggests the necessity for specific, culturally oriented prevention/intervention strategies with a view to reducing gender stereotypes and inequalities. Moreover, the large heterogeneity by age group observed underlines the need to develop primary and secondary prevention activities and for further studies on lethal risk factors across victim age groups. Cultural and educational contexts could provide several possible explanations for femicide among older women, as these deaths could be the fatal result of a long history of violence that was never recognized nor reported. It is also possible that some of these deaths could have been merciful, “as a solution to deteriorating health situations,” underlining the possible need to increase older people's social support levels and reduce their sense of isolation (28).

## Data Availability Statement

The data analyzed in this study is subject to the following licenses/restrictions: The data were retrieved within the project Socioeconomic differences in mortality (IF IST 2646), part of the National Statistical Program (PSN) approved by the Italian Data Protection Authority (13). All findings were produced using aggregated and not individual data. Requests to access these datasets should be directed to www.istat.it.

## Author Contributions

MV, AD, LF, MP, CM, and AP took part in the conceptualization of the study and preparation of the manuscript. MV, AD, LF, MP, and AP took part in the implementation of methods. MV, AD, and AP took part in bibliographic research, development, and statistical analysis. All the authors could access and verify the data, approve the manuscript, and are responsible for the views expressed in it.

## Conflict of Interest

The authors declare that the research was conducted in the absence of any commercial or financial relationships that could be construed as a potential conflict of interest.

## Publisher's Note

All claims expressed in this article are solely those of the authors and do not necessarily represent those of their affiliated organizations, or those of the publisher, the editors and the reviewers. Any product that may be evaluated in this article, or claim that may be made by its manufacturer, is not guaranteed or endorsed by the publisher.

## Supplementary Material

Supplementary Figure 1 Population at baseline and during the follow up.
