# Supplementary figures and images for: Male and Female Differences in Homicide Mortality: Results of an Italian Longitudinal Study, 2012–2018
Source: Front Public Health. 2022 Jul 13;10:919335. doi: 10.3389/fpubh.2022.919335 (PMC9326256; doi:10.3389/fpubh.2022.919335)

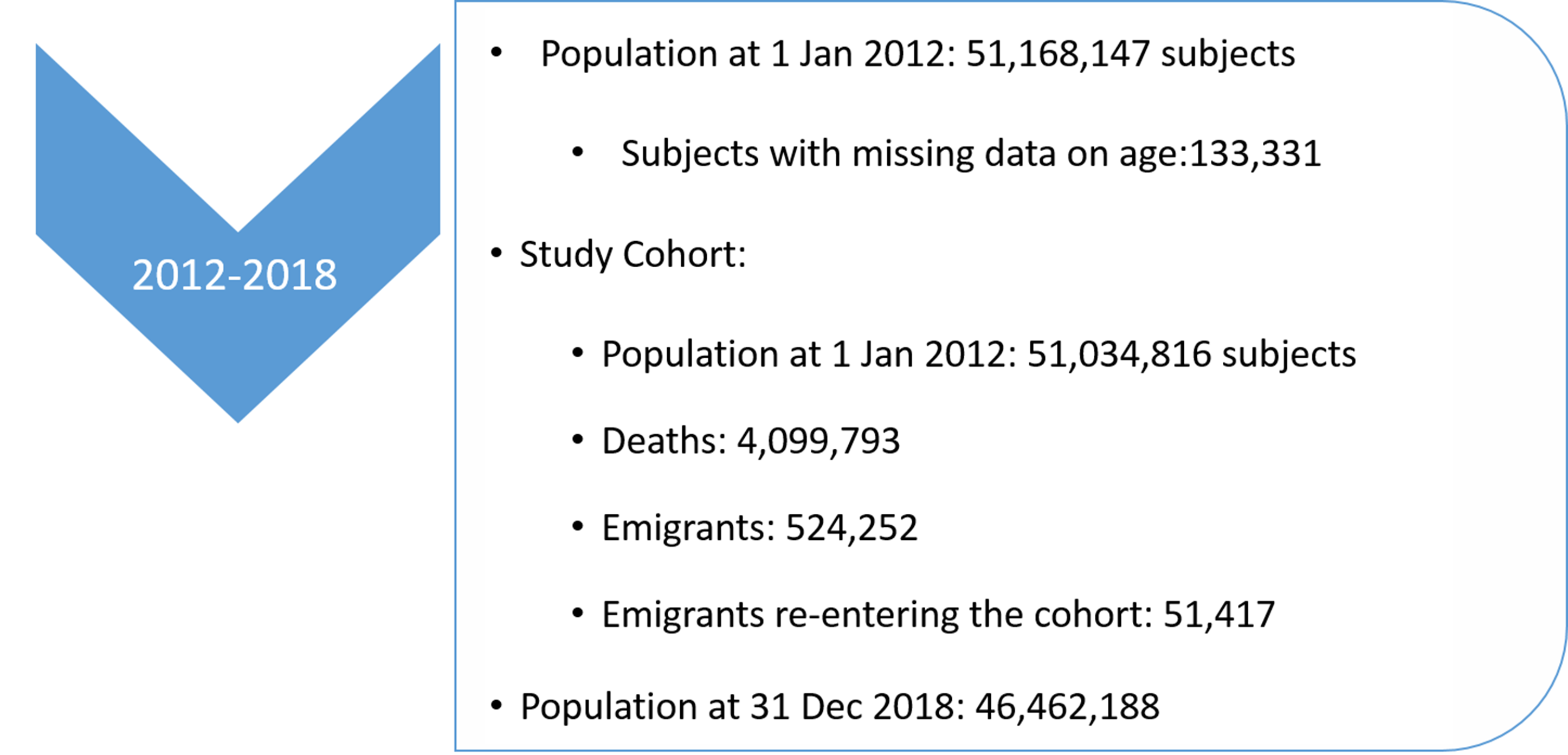

Supplement: Supplementary Figure 1 — Population at baseline and during the follow up. [file Image_1.PNG]
